# Solubility of Metal Precursors in Supercritical CO_2_: Measurements and Correlations

**DOI:** 10.3390/molecules30081660

**Published:** 2025-04-08

**Authors:** Marlene Crone, Michael Türk

**Affiliations:** Institute for Technical Thermodynamics, Karlsruhe Institute of Technology (KIT), Engler-Bunte-Ring 21, 76131 Karlsruhe, Germany

**Keywords:** solubility, Cu(acac)_2_, Cu(tmhd)_2_, Pd(acac)_2_, Pd(tmhd)_2_, Pt(acac)_2_, Pt(cod)me_2_, CO_2_, density-based models

## Abstract

Knowledge of the solubility of metal precursors in supercritical (sc) CO_2_ is a key factor for determining the best operation conditions for the synthesis of supported metallic nanoparticles. In this paper, new experimental solubility data of Cu(acac)_2_, Pd(acac)_2_, and Pt(acac)_2_ in scCO_2_ for temperatures from 313 to 353 K and pressures from 10 to 40 MPa are presented and compared with the literature data and correlated with semi-empirical density-based models (Chrastil, extended Kumar and Johnston, extended Bartle, and the original and modified Méndez–Santiago–Teja). In addition, literature data for the solubility of Cu(tmhd)_2_, Pd(tmhd)_2_, and Pt(cod)me_2_ in scCO_2_ were also correlated with the above-mentioned models. The best result, i.e., the best agreement between the experimental and calculated solubility datasets, was observed for the Chrastil model. Applying the Chrastil and extended Bartle models, the dissolution, sublimation, and solvation enthalpies were estimated. Furthermore, these correlation results were compared with the results from Ushiki et al., who correlated the solubilities of metal acetylacetonates in scCO_2_ from the literature using the PC-SAFT equation of state. This comparison showed that the original Méndez–Santiago–Teja model enabled a better description of the experimental data by a factor of three.

## 1. Introduction

The design, development, and synthesis of nanostructured materials, such as supported metal nanoparticles (NPs), are of particular interest to a large number of technologically important areas, such as chemistry, energy, electronics, optics, pharmacology, and material science. NPs are characterized by unique properties such as their high specific surface areas, leading to an enhanced energetic state and, thus, a higher reactivity. In particular, the synthesis of supported mono- or bimetallic NPs by supercritical fluid (SCFs)-based particle formation processes is a broad field of promising applications [1,2]. For example, in the case of particle formation processes such as a rapid expansion of supercritical solutions, supercritical fluid reactive deposition (SFRD), etc., the properties of the produced particles, such as particle size and morphology [3], dissolution behavior [4] or catalytic activity [5], are often strongly influenced by the underlying phase behavior.

A promising synthesis route is the use of supercritical (sc) CO_2_ as solvent, reaction, and separation media to synthesize nanostructured materials by SFRD. Numerous experimental results published in the literature demonstrate impressively that supported mono- or bimetallic NPs prepared by SFRD exhibit catalytic behavior that is much higher than the reference samples prepared by conventional methods (see, e.g., overview given in [2,6]). From a more general point of view, the solubility behavior affects the uptake and the size of the NPs [7] synthesized by SFRD and, thus, the catalytic activity of the supported NPs [2]. Thus, knowledge of metal precursor solubility in scCO_2_ is crucial for the determination of the best operating conditions since an insufficient solubility of the precursor in scCO_2_ limits practical applicability.

The literature contains numerous semi-empirical models for correlating the solubility of solids in scCO_2_ (e.g., [8,9]). These solubility data were correlated using two models in which the solubility of the dissolved solid, $y_{2}$, depends on the CO_2_ density and temperature (i.e., Chrastil [10] and Kumar and Johnston [11]), two models that describe solubility as a function of CO_2_ density, temperature, and pressure (i.e., Bartle et al. [12] and the modified Méndez-Santiago and Teja method [13]), and one model which describes solubility as a function of CO_2_ density, temperature, pressure, and sublimation pressure of the solid (i.e., the original Méndez-Santiago and Teja [13]). The parameters for these models (except for the latter model) were obtained by performing a multiple linear regression on the experimental solubility data using the software OriginPro^®^ 2022b. More details about these models are given in the corresponding original literature.

On the other hand, applying the cubic equations of state (EoS) requires knowledge of the thermophysical properties of the substances involved (such as critical properties and acentric factors) and at least one characteristic binary interaction parameter. However, due to partially low thermal degradation temperatures, reliable experimental critical data on the precursors are often not available in the literature, and the estimation techniques are inaccurate, as the contribution of metal cannot be taken into account [14]. Independently of whether the EoS approach or the enhancement factor E model [15,16] is applied, knowledge of the sublimation pressure is required for the correlation of solubility data [17]. Therefore, the influence of the sublimation pressure on the solubility correlation was also investigated.

## 2. Results

### 2.1. Presentation of the Models Used

#### 2.1.1. Density-Based Approaches

According to the review written by Knez et al. [9], a widely used semi-empirical model was developed by Chrastil more than 40 years ago [10]. As pointed out by Stiver [18], there have been a number of modifications to Chrastil’s model in an effort to improve the performance of the original equation [19,20,21]. Since most solubility data are reported on a mole fraction basis, Stiver suggested transferring Chrastil’s original equation with solubility units ($g\cdot {dm}^{-3}$) into an equation with solubility units of ($mol\cdot {mol}^{-1}$).

(1) $$ln\left(y_{2}\right)=\beta +\gamma \cdot ln\left(\frac{\rho_{1}}{\rho_{0}}\right)+\frac{\alpha }{T}$$

In Equation (1), $y_{2}$ is the solubility $mol\cdot {mol}^{-1}$; the density of pure CO_2_ is expressed as the normalized density $\rho_{1}/\rho_{0}\;with\;\rho_{0}=1\;mol\cdot {dm}^{-3}$; T is the temperature (K); and the three empirical Chrastil parameters are denoted by Greek letters (α, β, and γ) to distinguish the parameters of the modified equation from those of its original form [18]. The parameter γ expresses an average equilibrium association number, which is characteristic of a given binary system and represents the average number of CO_2_ molecules in an assumed solvent–solute complex. α (K) is a parameter that is a function of the enthalpy of dissolution, ${\Delta h}_{diss}\;\left(kJ\cdot {mol}^{-1}\right)$, which is the sum of the enthalpies of sublimation (or vaporization), ${\Delta h}_{sub}$, and solvation of the solute, ${\Delta h}_{solv}$, while β depends on the molecular weights of the solute and solvent. Furthermore, Equation (1) follows that plots of $\left[ln\left(y_{2}\right)-\left(\alpha /T\right)\right]\;vs.\;ln\left(\rho_{1}/\rho_{0}\right)$ for different temperatures will collapse to a single straight line, and the slope is equal to γ.

The widely used modified approach from Kumar and Johnston (KJ ext.) proposed a semi-empirical model in which the solubility of the dissolved solid depends on the pure CO_2_ density and system temperature [11,22]:

(2) $$ln\left(y_{2}\right)=b_{0}^{⋆}+b_{1}^{⋆}\cdot \rho_{1}+\frac{b_{2}^{⋆}}{T}$$

In Equation (2), $b_{0\;}^{⋆}$ is the intersection with the y-axis; the slope $b_{1}^{⋆}\;\left({dm}^{3}\cdot {mol}^{-1}\right)$ is related to the solvent isothermal compressibility and the partial molar volume of the solute present at an infinite dilution in the SCF phase; and the value of $b_{2}^{⋆}$ (K) is related to ${\Delta h}_{diss}$. Again, plots of $\left[ln\left(y_{2}\right)-b_{2}^{⋆}/T\right]\;vs.\;{\;\rho }_{1}$ for different temperatures should collapse to a single straight line, and the slope is equal to $b_{1}^{⋆}$.

Based on a model suggested by Bartle et al. [12], Miller et al. [23] proposed an extended model (Bartle ext.) that correlates the solubility $y_{2}$ with the solvent’s density in the following way:

(3) $$ln\left(\frac{y_{2}\cdot p}{p_{ref}}\right)=a_{0}+a_{1}\cdot \left(\rho_{1}-\rho_{ref}\right)+\frac{a_{2}}{T}$$

In Equation (3), $p_{ref}=0.1\;MPa$ is the reference pressure, and $\rho_{ref}=15.90\;mol\cdot {dm}^{-3}$ is the reference density; $a_{0},$ $a_{1}\;\left({dm}^{3}\cdot {mol}^{-1}\right)\;and\;a_{2}\;\left(K\right)$ are the model’s parameters. Upon a suggestion by Miller et al., parameter $a_{2}$ can approximately be related to the sublimation enthalpy of the solid ${\Delta h}_{sub}$ [23]. It should be noted that the sublimation enthalpy estimated using this method is an approximate value. Plots of $\left[ln\left(y_{2}\cdot p/p_{ref}\right)-a_{2}/T\right]\;vs.\;\left(\rho_{1\;}-\rho_{ref}\right)$ for different temperatures should collapse to a single straight line, and the slope is equal to $a_{1}$.

One of the most commonly used models, which correlates the solubility $y_{2}$ of a solid in an SCF to the pure fluid’s density has been proposed by Méndez-Santiago and Teja (MST) [13]. This semi-empirical model (MST orig.) is based on the theory of dilute solutions. The linear relationship is as follows:

(4) $$T\cdot ln\left(E\right)=A+B\cdot \rho_{1}$$

This can be used to prove the consistency of the experimental data. In Equation (4), $\rho_{1}$ is the density of pure CO_2_ at the equilibrium temperature T and pressure p. The so-called enhancement factor *E* is defined as the ratio of the mole fraction of the solid over the solubility in an ideal gas as follows:

(5) $$E=\frac{y_{2}\cdot p}{p_{2,sub}(T)}$$

E can be interpreted as a normalized solubility since it eliminates the effect of the solid’s sublimation pressure $p_{2,sub}\;(Pa)$ at temperature T.

The two parameters from Equation (4), A $and\;B\;\left({dm}^{3}\cdot {mol}^{-1}\right)$, are independent of the temperature so that the solubility data for binary systems at different temperatures should collapse to a single straight line when plotted $\left[\;T\cdot ln\left(E\right)\right]\;vs.\rho_{1}$ and the slope is equal to B.

For the calculation of the enhancement factor E, reliable sublimation pressure data are required. Since such experimental data for solids are often not available, Méndez–Santiago and Teja incorporated the Clausius–Clapeyron-type expression for the sublimation pressure in Equation (6):

(6) $$ln\left(\frac{p_{2,sub}\left(T\right)}{p_{0}}\right)=a+\frac{b}{T}$$

where $p_{0}=1\;Pa$ is the reference pressure, and a and $b\;\left(K\right)$ are the empirical parameters that are fitted to the experimental sublimation pressure data. As a result, Méndez–Santiago and Teja modified Equation (4) and proposed a semi-empirical correlation for the solid solubility, which has three adjustable parameters, $A^{⋆},\;B^{⋆}\;and\;C^{⋆}$ (MST mod.), which are independent of the temperature and pressure [13]:

(7) $$\ln\left(y_{2}\cdot \frac{p}{p_{\#}}\right)=\frac{A^{⋆}}{T}+\frac{B^{⋆}\cdot \rho_{1}}{T}+C^{⋆}$$

Similar to the model proposed by Miller et al. [23], the normalized pressure $p/p_{\#}\;with\;p_{\#}=1\;MPa$ is incorporated into Equation (7). $A^{⋆}\left(K\right),\;B^{⋆}\;\left({K\cdot dm}^{3}\cdot {mol}^{-1}\right)\;\mathrm{and}\;C^{⋆}$ are model parameters. Furthermore, the fact that all isotherms should collapse to a single line when plotted $\left[T\cdot \left[\ln\left(y_{2}\cdot p/p_{\#}\right)-C^{⋆}\right]\right]\;vs.\rho_{1}$ allows for determining the consistency of different experimental datasets.

It is worth noting that density-based approaches, which are based on the theory of dilute solutions, have some limitations. If the solubility is high (appr. 0.035 $mol\cdot {mol}^{-1}$), the pure solvent density has to be corrected since the density of the binary system differs significantly from that of the pure solvent [10,13,18]. Also, these density-based approaches are only valid over a limited range of temperature and pressure. It is important to consider that the upper limit of this linear behavior is about twofold, while the lower limit is around 0.6 of the solvent’s critical density [13] with $\rho_{c}=10.5\;mol\cdot {dm}^{-3}$ [24].

Furthermore, not all experimental datasets are suitable for the Chrastil, KJ ext., Bartle ext., or MST mod. approaches (Equations (1), (2), (3), and (7)) since datasets with less than five data points [18] are not applicable for fitting a model with three independent variables. Furthermore, isothermal data, i.e., measured at one single temperature, are inadequate to describe the temperature dependence. Nevertheless, these correlations are very useful because knowledge regarding the thermophysical properties of the solid (e.g., critical temperature, critical pressure, acentric factor, and sublimation pressure) is not necessary.

#### 2.1.2. Equation of State Approaches

In addition to the density-based models, an approach based on cubic EoS is frequently used for the calculation of solid solubility in scCO_2_ as a function of temperature and pressure. Based on the equifugacity condition between the solid and fluid phases, this approach requires not only selecting the most appropriate EoS and mixing rule but also knowledge of the pure component parameters of both the fluid and the solid. Thus, modeling the solubility behavior by cubic EoS is a challenging task [22,25,26]. In principle, this challenge is a twofold one, namely both to correlate existing data and the prediction of phase equilibria in regions where experimental data are not yet available.

Pressure explicit EoS generally expresses the pressure p as the sum of two terms, a repulsion pressure $p_{R}$ and an attraction pressure $p_{A}$, as shown in Equation (8) [27,28]:

(8) $$p=p_{R}+p_{A}$$

Assuming that the molar volume of the pure solid ($v_{2,s}$) at the system temperature is pressure independent, $y_{2}$ can be calculated according to Equation (9) as follows [29]:

(9) $$y_{2}\left(T,p\right)=\frac{\phi_{2,s}\left(T,p_{2,sub}\left(T\right)\right)\cdot p_{2,sub}\left(T\right)}{\phi_{2}\left(T,p,y_{2}\right)\cdot p}\cdot exp\left[\frac{v_{2,s}\cdot \left(p-p_{2,sub}\left(T\right)\right)}{R_{0}\cdot T}\right]$$

where $p_{2,sub}$ is the sublimation pressure at temperature T, and $\phi_{2,s}$ is the fugacity coefficient at T and $p_{2,sub}$, which is equal to one due to the low sublimation pressure of solids. $\phi_{2}$ is the fugacity coefficient of the solid in the SCF phase at the current $T,p,y_{2}$, and $R_{0}$ is the universal gas constant. Among others, the well-known Peng–Robinson EoS (PR EoS), in combination with the van der Waals-1 mixing rules using one, $k_{ij}$, [27] or two binary interaction parameters, $k_{ij}$ and $l_{ij}$, [29] are often used for the calculation of $\phi_{2}$.

Recently, the perturbed-chain statistical associating fluid theory (PC-SAFT) EoS was used by Ushiki et al. to calculate the solubilities of metal precursors in scCO_2_ [14,30]. In short, the PC-SAFT EoS describes the molar residual Helmholtz energy $A_{res}$ of a system as the sum of different additive and independent contributions, as shown in Equation (10):

(10) $$A_{res}=A_{hc}+A_{disp}+A_{assoc}$$

These contributions are the hard-chain repulsion contribution $A_{hc}$ and the dispersion (van der Waals) attraction contribution $A_{disp}$. Since associating components that form hydrogen bonds do not exist in the examined systems, the contribution of the molecular association $A_{assoc}$ was not considered by Ushiki et al. For the description of the phase behavior of a mixture composed of components i and j, the conventional Lorentz–Berthelot combining rules were usually applied [31]. Thereby, the binary interaction parameter $k_{12}$, which describes the interactions between the components i and j, was used as a fitting parameter. More details about this approach can be found in the literature [32].

#### 2.1.3. Evaluation of the Fitting Quality

The squared correlation coefficient ($R^{2}$) and the adjusted $R^{2}$ ($R_{adj}^{2}$) are used for statistical analysis [33]. As a rule of thumb, the more parameters there are for curve fitting, the more accurate the correlations are in order to obtain a reliable accuracy criterion for comparing the different models with a different number of fitting parameters. $R^{2}$ in Equation (11) is expressed as:

(11) $$R^{2}={\left[\frac{\sum_{i=1}^{n}\left(y_{2}^{exp}-\bar{y_{2}^{exp}}\right)\cdot \left(y_{2}^{calc}-\bar{y_{2}^{calc}}\right)}{\sqrt{\sum_{i=1}^{n}{\left(y_{2}^{exp}-\bar{y_{2}^{exp}}\right)}^{2}}\cdot \sqrt{\sum_{i=1}^{n}{\left(y_{2}^{calc}-\bar{y_{2}^{calc}}\right)}^{2}}}\right]}^{2}$$

where $y_{2}^{calc}$ and $y_{2}^{exp}$ are the calculated and experimental solubility for each experimental data point; $\bar{y_{2}^{exp}}$ and $\bar{y_{2}^{calc}}$ are the global mean values of the experimental and calculated data. Note that a $R^{2}$ value of 1 means that the agreement between the calculated and experimental data is perfect.

The $R_{adj}^{2}$ value is a modification of $R^{2}$ and enables the comparison of models with different adjustable parameters and can have negative, less than, or equal to $R^{2}$ values. The frequently used adjusted $R_{adj}^{2}$ in Equation (12) is expressed as:

(12) $$R_{adj}^{2}=1-\frac{N-1}{N-K}\cdot \left(1-R^{2}\right)$$

where $R^{2}$ is the coefficient of determination, N is the number of experimental data, and K the number of adjustable parameters in the correlation model. Since $R_{adj}^{2}$ considers the number of adjustable parameters, this value can be used to compare models with different numbers of independent variables. However, it must be noted that in the case of a large number of data, this correction is negligible.

For each of the above models, the average absolute relative deviation AARD (%) between the experimental and modeled data for $y_{2}$ were calculated according to Equation (13) and used to evaluate the accuracy of the different models:

(13) $$AARD=\left(\frac{1}{N}\sum_{1}^{N}\frac{\left|y_{2}^{calc}-y_{2}^{exp}\right|}{y_{2}^{exp}}\right)\cdot 100$$

where N is the number of data points.

### 2.2. Experimental Solubility Data

The solubility of the precursor in CO_2_ can be calculated from the dissolved molar amount of precursor divided by the sum of the dissolved molar amount of precursor plus the molar amount of CO_2_ dosed into the view cell (see Equation (14)):

(14) $$y_{2}=\frac{n_{2}}{n_{2}+n_{1}}$$

with $n_{i}=m_{i}/M_{i}$, where $n_{1}$ and $n_{2}$ are the moles of CO_2_ and the precursor, respectively, $m_{i}\;(g)$ is the corresponding masses, and $M_{i}$ $\left(g\cdot {mol}^{-1}\right)$ is the corresponding molar masses.

The experimental data for the solubility of copper (II) acetylacetonate (Cu(acac)_2_), palladium (II) acetylacetonate (Pd(acac)_2_), and platinum (II) acetylacetonate (Pt(acac)_2_) in scCO_2_ were determined at different temperatures and pressures and are summarized in Table 1, Table 2 and Table 3. The values listed in Table 1, Table 2 and Table 3 for ${\Delta \rho }_{1}/\rho_{1}$ and ${\Delta y}_{2}$ are calculated on the basis of the individual uncertainties for pressure, temperature, and mass determination, which are discussed in Section 3.2.

For the scCO_2_/Cu(acac)_2_ system, the solubility increases at 333 K and 15.2 MPa from $y_{2}$ = 1.05·10^−5^ to 3.16·10^−5^ $mol\cdot {mol}^{-1}$ at 40.1 MPa, and at 353 K and 15.1 MPa from $y_{2}$ = 9.66·10^−6^ to 5.96·10^−5^ at 40.5 MPa. The solubility of Pd(acac)_2_ in scCO_2_ increases at 313 K and 10.1 MPa from $y_{2}$ = 8.95·10^−6^ to 2.30·10^−5^ at 30.1 MPa, and at 353 K and 10.1 MPa from $y_{2}$ = 9.13·10^−6^ to 9.92·10^−5^ at 29.8 MPa. For the scCO_2_/Pt(acac)_2_ system, the solubility increases from 313 K and 15.2 MPa from $y_{2}$ = 1.37·10^−5^ to 2.88·10^−5^ at 40.1 MPa, and at 353 K and 15.2 MPa from $y_{2}$ = 7.34·10^−6^ to 1.22·10^−4^ at 40.2 MPa. At a given temperature and pressure condition, the order of the solubility of the precursors in scCO_2_ is: Pd(acac)_2_ > Pt(acac)_2_ > Cu(acac)_2_. The comparison of these (acac)_2_ precursors with the experimental solubility data at 353 K and 30 MPa published for Cu(tmhd)_2_ and Pt(cod)me_2_ [34] show that these solubility data are by a factor of about 70 higher, while the solubility data published for Pd(tmhd)_2_ [35] are by a factor of about 14 higher.

For all systems investigated, it holds that for a given pressure, the solubility of the precursors in scCO_2_ decreases with increasing temperatures for pressures between the lower and the upper crossover pressures (retrograde region) [36]. Outside this region, the opposite behavior is observed. Note that the upper crossover pressure for the systems investigated was found to be around 16 MPa and is caused by the competition between the impact of the precursor sublimation pressure and the CO_2_ density on temperature.

### 2.3. Experimental Sublimation Pressure Data from the Literature

**Figure 1:**
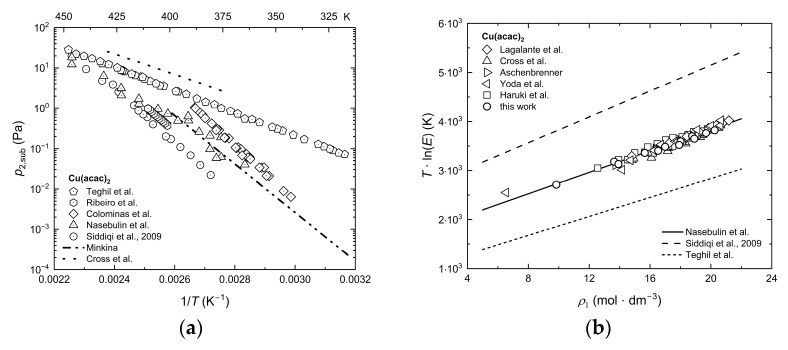
For the CO_2_/Cu(acac)_2_ system: (**a**) experimental sublimation pressure data available in the literature [37,38,39,40,41,42,43]; (**b**) $T\cdot ln\left(E\right)$ vs. CO_2_ density [37,39,40,44,45,46,47].

For certain density-based approaches, e.g., MST orig., experimental sublimation pressure data, $p_{2,sub}$, are required. Figure 1a shows for Cu(acac)_2_ the numerous available experimental sublimation pressure data from the literature; at low temperatures (around 325 K), the differences for $p_{2,sub}$ are up to three orders of magnitude, but they become significantly smaller with an increasing temperature.

The influence of the sublimation pressure on the enhancement factor E is clarified exemplarily for Cu(acac)_2_ in Figure 1b. It is obvious that the majority of the Cu(acac)_2_ solubility data published by different authors collapse to a single line if the experimental sublimation pressure data from Nasebulin [37] are used to calculate E. On the opposite thereto, the data published by Siddiqi [38] led to significantly higher values, while the data published by Teghil [39] led to clearly lower values for the enhancement factor E. This course is due to the noticeably higher values from Teghil [39] and the lower values from Siddiqi [38] published for $p_{2,sub}$ (cf. Figure 1a). Therefore, when selecting the data for $p_{2,sub}$ for the consistency test for the precursors investigated, the extent to which they match the solubility data was first checked.

It follows from the results obtained that the experimental sublimation pressure data are subject to large uncertainties and must, therefore, very often be estimated by using empirical group contribution methods (GCM). However, the common GC methods cannot be applied to metal precursors because the contribution of the metal atom cannot be considered [14]. Similar to the results shown in Figure 1b, it was also shown in [17] that the use of different GCM methods also leads to significantly different values for E.

In summary and in opposite to the results obtained for Cu(acac)_2_, Pd(acac)_2_, Pt(acac)_2_, Cu(tmhd)_2_, and Pd(tmhd)_2_, the different experimental data for Pt(cod)me_2_ do not collapse to a single line when using the experimental. $p_{2,sub}$ data from Hierso et al. [48], which are, to our knowledge, the only $p_{2,sub}$ data published in the literature. Thus, in the case of Pt(cod)me_2_, MST orig. was not able to prove the consistency of the different experimental solubility data (cf. Figure A4 in Appendix B and [34]).

### 2.4. Modeling of Solubility Data

The reliable experimental determination of the solubility in SCFs and its accurate modeling are important for the development of supercritical fluid technology. Therefore, different empirical density-based models were used for the correlation of experimental solubility data. In addition to our own solubility data (cf. Table 1, Table 2 and Table 3), experimental data from the literature (cf. Table 4) were used for modeling the solubility behavior of the (acac)_2_ precursors and of Cu(tmhd)_2_, Pd(tmhd)_2_, and Pt(cod)me_2_ in scCO_2_.

The linear relationships of Equations (1), (2), (3), (4), and (7) enable identifying experimental solubility data that are not consistent with other data. For most of the datasets (20 of 22) investigated, it holds that a pronounced linear trend was observed for a density range starting around 6.3 $mol\cdot {dm}^{-3}$ (about 0.6·$\rho_{c}$) to nearly 22 $mol\cdot {dm}^{-3}$ (about twofold of $\rho_{c}$ of CO_2_), which represents the upper limit of the available experimental data.

The values obtained from fitting the individual model parameters of Equations (1), (2), (3), (4), and (7), along with the $R_{adj}^{2}$ values for our own and literature solubility data are summarized in Table 5.

**Figure 2:**
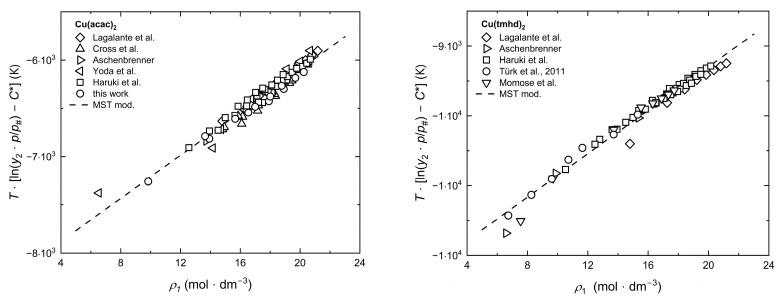

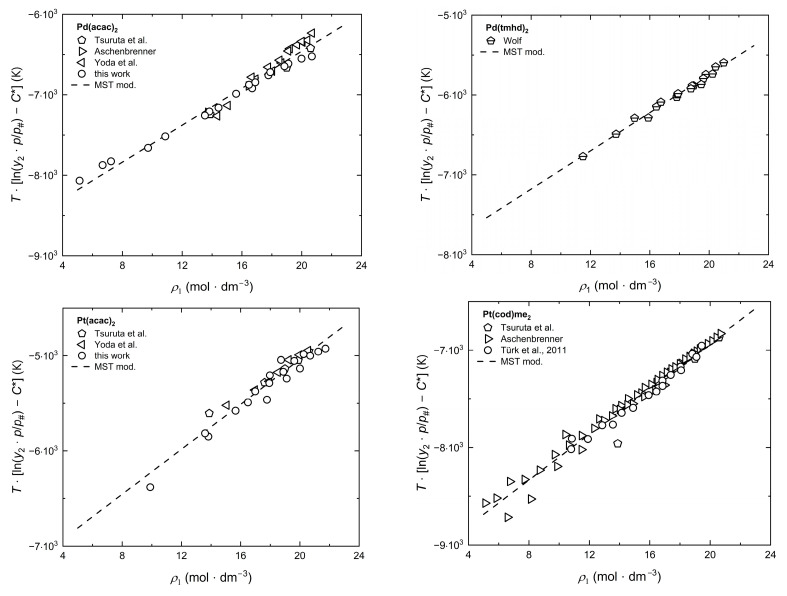
Consistency test for the solubility of different precursors in scCO_2_ using the MST mod. model [34,35,40,44,45,46,47,49,50].

**Figure A1:**
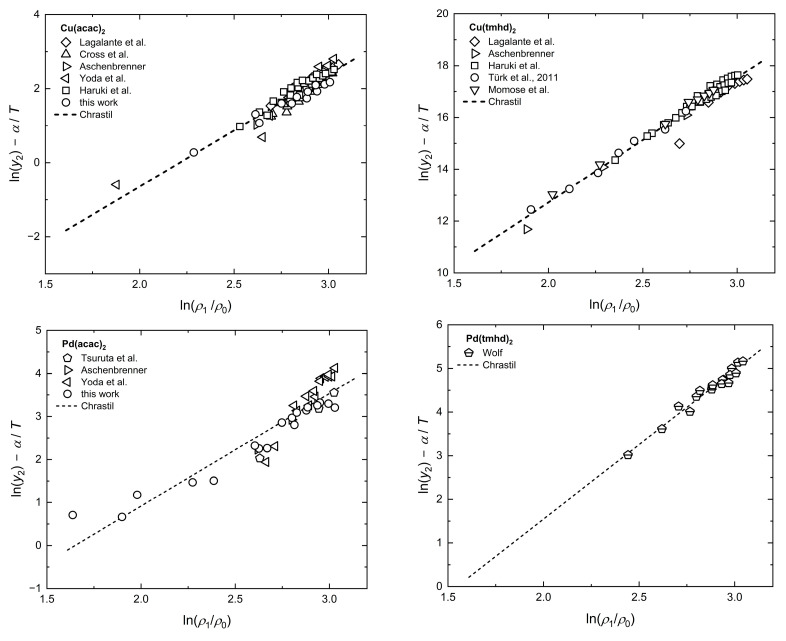

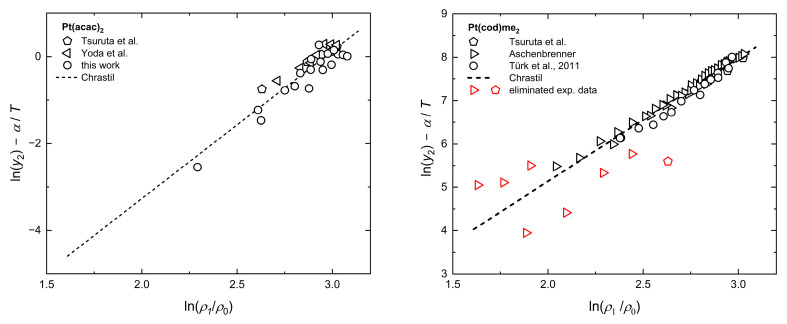
Consistency test for the solubility of different precursors in scCO_2_ using the Chrastil model [34,35,40,44,45,46,47,49,50].

**Figure A2:**
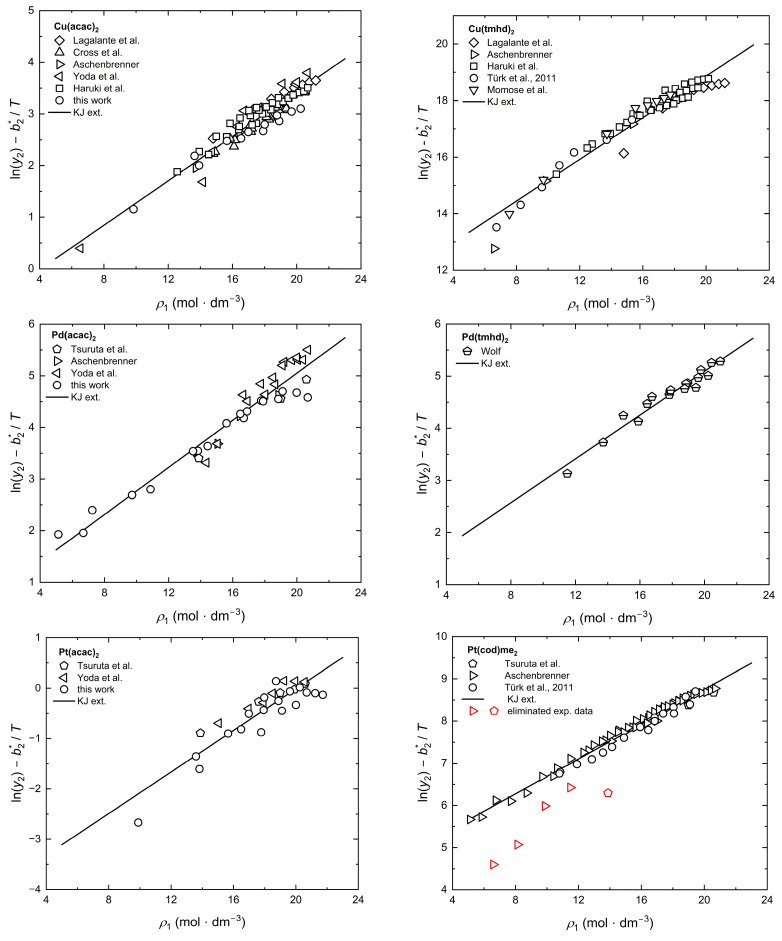
Consistency test for the solubility of different precursors in scCO_2_ using the KJ ext. model [34,35,40,44,45,46,47,49,50].

**Figure A3:**
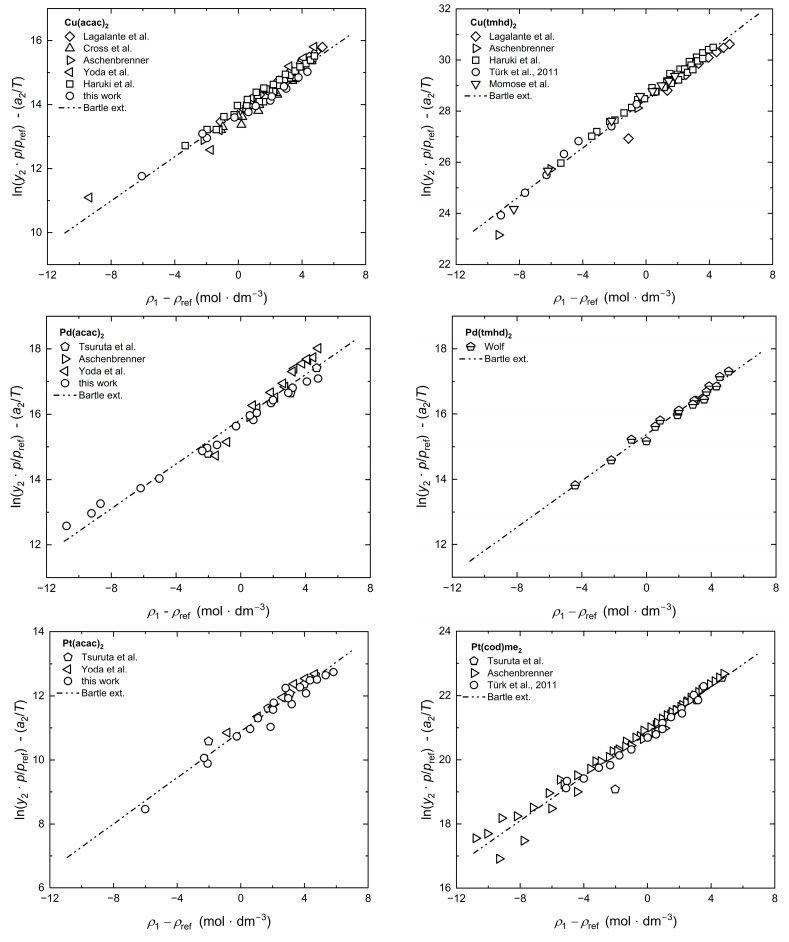
Consistency test for the solubility of different precursors in scCO_2_ using the Bartle ext. model [34,35,40,44,45,46,47,49,50].

**Figure A4:**
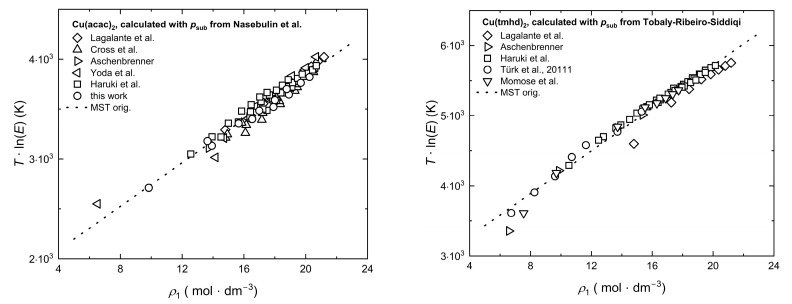

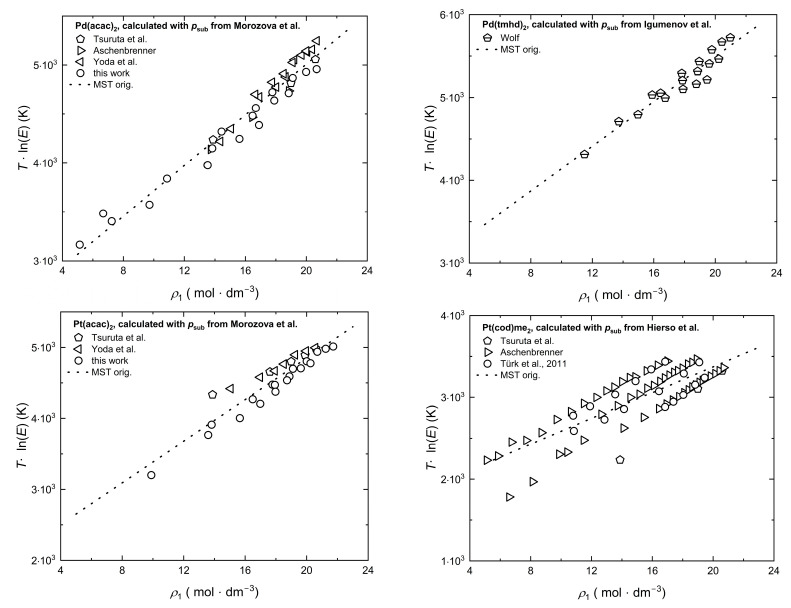
Consistency test for the solubility of different precursors in scCO_2_ using the MST orig. model [34,35,40,44,45,46,47,49,50].

Figure 2 shows exemplary results for the consistency tests with MST mod. For the sake of completeness, the consistency tests with Chrastil, KJ ext., Bartle ext., and MST orig. are depicted in Figure A1, Figure A2, Figure A3 and Figure A4 in Appendix B. From these figures, the model approach and the experimental solubility data confirm the consistency of the experimental solubility data across all temperatures.

**Figure 3:**
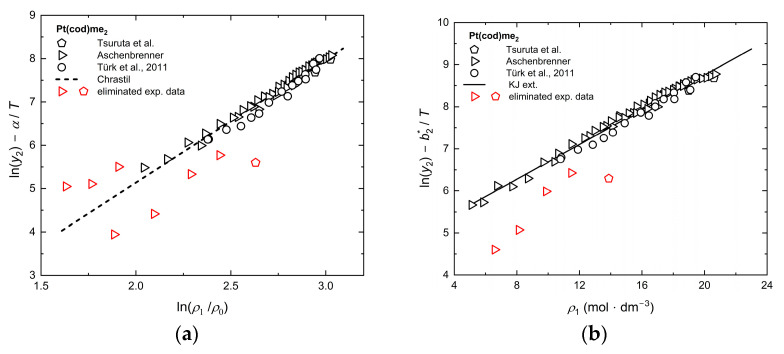
Consistency test (**a**) with the Chrastil model and (**b**) with the KJ ext. model [34,45,50]. The red symbols identify experimental data points that failed the thermodynamic consistency test and were, therefore, eliminated.

As shown in Figure 3 for the Chrastil (a) and KJ ext. (b) models, significant deviations from the linear trend were observed for some solubility data from Aschenbrenner [45] and Tsuruta et al. [50] for Pt(cod)me_2_ in scCO_2_. Therefore, the data at 333 K and 10 MPa, 11 MPa, 12 MPa, and 13 MPa, as well as at 313 K and 9.8 MPa, were excluded for fitting the model parameters of KJ ext., and additionally, the data at 353 K and 10 MPa and 11 MPa and 12 MPa were excluded for fitting the Chrastil model. This improved the $R_{adj}^{2}$ value for the KJ ext. approach from 0.93 to 0.98, and for Chrastil, from 0.90 to 0.97.

**Figure 4:**
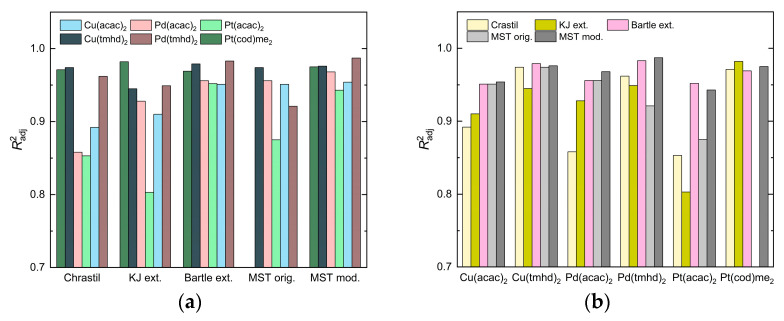
Comparison of the $R_{adj}^{2}$ values calculated for the density-based approaches: (**a**) plotted over these approaches and (**b**) plotted over the precursors investigated.

Table 5 and Figure 4 shows that the $R_{adj}^{2}$ values range from 0.85 to 0.97 for Chrastil, from 0.80 to 0.98 for KJ ext., from 0.95 to 0.98 for Bartle ext., from 0.88 to 0.97 for MST orig., and from 0.94 to 0.99 for MST mod. Thus, the best fit of the respective model parameters was achieved with Bartle ext. and MST mod. Among the different precursors examined, the best fit was received for Cu(tmhd)_2_ and Pd(tmhd)_2_ when applying the Bartle ext. and MST mod. approaches. For the three (acac)_2_ precursors, the Chrastil approach yields the lowest $R_{adj}^{2}$ values (<0.9), while the highest $R_{adj}^{2}$ values were obtained for Cu(tmhd)_2_, Pd(tmhd)_2_, and Pt(cod)me_2_ (>0.96).

### 2.5. Correlation of Experimental Solubility Data

**Figure 5:**
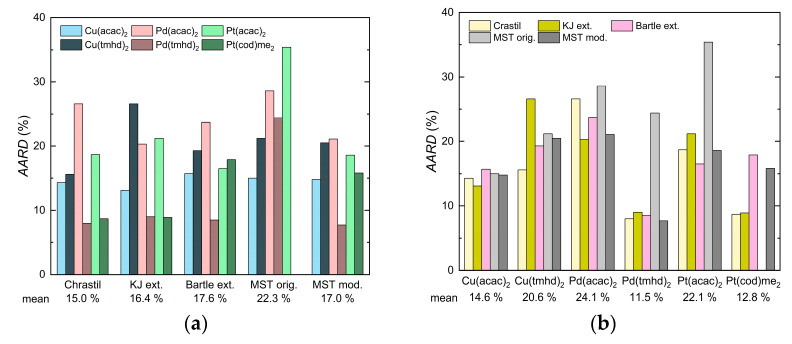
Comparison of the AARD values between different density-based approaches used in this investigation: (**a**) plotted over these approaches and (**b**) plotted over the precursors investigated.

The results for modeling the solubility behavior of the various precursors in scCO_2_ with Equations (1)–(4) and (7) are summarized in Table 6 along with the AARD values and are depicted in Figure 5. This shows that the mean overall AARD values are 15.0% for Chrastil, 16.4% for KJ ext., 17.6% for Bartle ext., 22.3% for MST orig., and 17.0% for MST mod. Looking at the individual precursors, the mean overall AARD values are 14.6%, 20.6%, 24.1%, 11.5%, 22.1%, and 12.8% for Cu(acac)_2_, Cu(tmhd)_2_, Pd(acac)_2_, Pd(tmhd)_2_, Pt(acac)_2_, and Pt(cod)me_2_, respectively.

In order to interpret these results with regard to the required system (ρ,T,p) and solid ($p_{2,sub}$) properties in more detail, the following must be taken into account: in the case of Chrastil and KJ ext., only the density and temperature data are required, i.e., $y_{2}=f\left(\rho ,\;T\right)$, while for Bartle ext. and MST mod., in addition to the density and temperature, the pressure is required, i.e., $y_{2}=f\left(\rho ,T,p\right)$. Moreover, for MST orig., the sublimation pressure is needed, i.e., $y_{2}=f\left(\rho ,T,p,\;p_{2,sub}\right)$.

In summary, it can be said that the Chrastil and KJ ext. models show better agreement between the experimental and calculated solubility data than the Bartle ext., MST mod., and MST orig. models. It should also be noted that, as pointed out by Kautz et al. in detail, the introduction of a fourth adjustable parameter does not significantly improve the correlation [25]. In addition, the quality of the data representation seems to decrease when, as in the case of Bartle ext. and MST mod., the pressure is included. Thus, Chrastil’s simple model leads to the best results and remains, therefore, a reliable model for describing the solubility of such precursors in scCO_2_.

**Figure 6:**
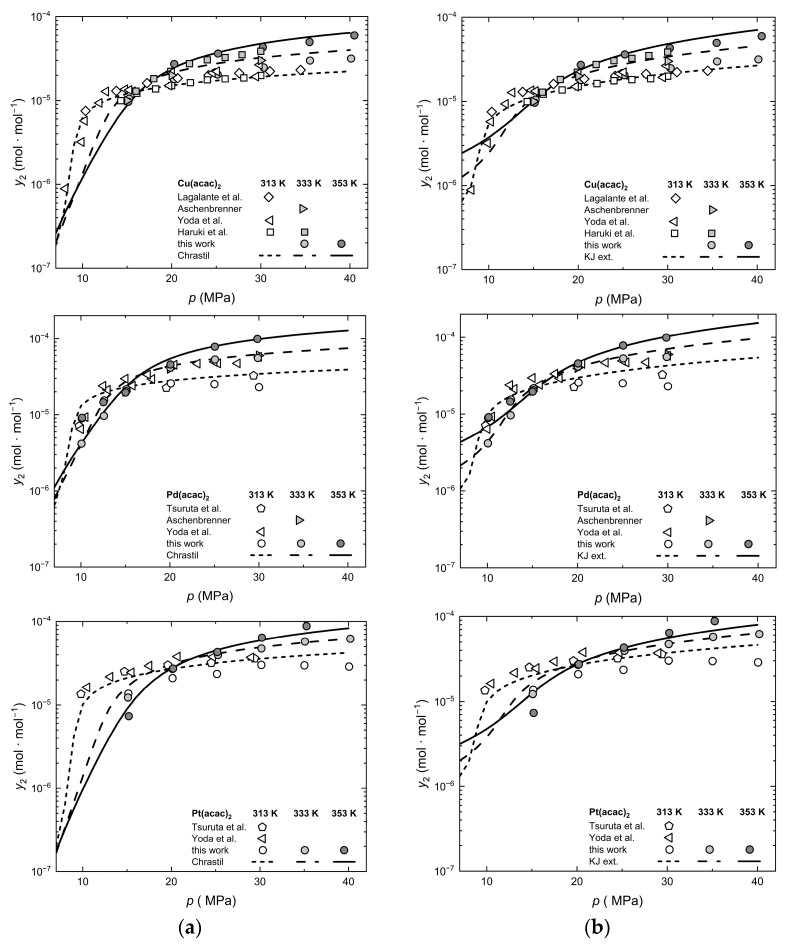
Experimental solubility data for Cu(acac)_2_, Pd(acac)_2_, and Pt(acac)_2_ [44,45,46,47,50], (**a**) calculated with Chrastil and (**b**) calculated with KJ ext.

Since the measured and controlled property in technical processes is pressure rather than density, it is important to have information regarding the influence of pressure and temperature on solubility $y_{2}$. The correlation results obtained with the Chrastil and KJ ext. models are depicted in Figure 6, which shows solubility $y_{2}$ as a function of pressure at different temperatures for the three (acac)_2_ precursors.

Cross et al. showed that the PR EoS, in combination with the van der Waals-1 mixing rules, is able to correlate the Cu(acac)_2_ solubility data in the range from 308 to 328 K and a CO_2_ density up to 0.9 $g\cdot {cm}^{-3}$ (=20 $mol\cdot {dm}^{-3}$, respectively) well if $k_{ij}$ is modeled as a temperature-dependent interaction coefficient [40].

Upper used the PR EoS together with the van der Waals-1 mixing rules to correlate the solubility of Pt(cod)me_2_ in scCO_2_, and the obtained AARD values were 6.4% at 313 K, 12.7% at 333 K, and 21.4% at 353 K. However, significantly greater deviations were observed when using the Redlich–Kwong–Soave EoS [26].

Ushiki and coworkers applied the PC-SAFT EoS for modeling the solubility of, among others, six (acac)_x_ precursors in scCO_2_ [14,30], published in the literature for Cu(acac)_2_, Pd(acac)_2_, and Pt(acac)_2_ [46,47]. The pure component parameters (i.e., the segment diameter, segment number, and dispersion energy) for the different precursors were determined by fitting experimental solubility data in organic solvents. Thereby, the binary interaction parameter $k_{ij}$ was set to zero. The pure component parameters determined in this way were then used to calculate the solubility of the precursors in scCO_2_ for each isotherm and compared with the experimental data. Thereby, it must be noted that for Cu(acac)_2_ as well as Pd(acac)_2_ and Pt(acac)_2_, the individual $k_{ij}$ values were adjusted isothermally [14,30]. In contrast to the results for the Cu(acac)_2_, Pd(acac)_2_, and Pt(acac)_2_ given in Table 6, the AARD values for MST orig., given below, were obtained by isothermal fitting in order to compare them directly with the PC-SAFT results from Ushiki et al.

**Figure 7:**
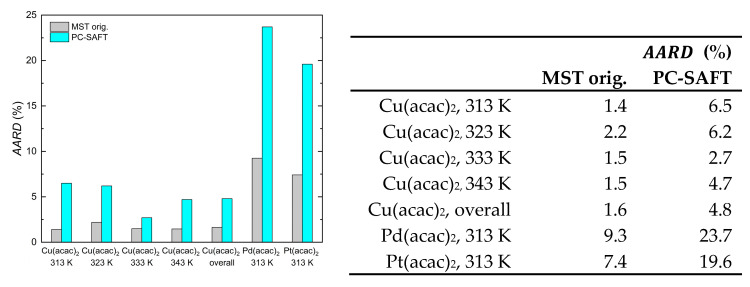
Comparison of the AARD values calculated for each isotherm with MST orig. and PC-SAFT EoS for Cu(acac)_2_ [30], Pd(acac)_2_, and Pt(acac)_2_ [14].

In the case of PC-SAFT, AARD values of 4.8% for Cu(acac)_2_, 24% for Pd(acac)_2_, and 20% for Pt(acac)_2_ were achieved. The detailed comparison of the different AARD values obtained from calculating the solubility data, either with the MST orig. model or the PC-SAFT EoS, is illustrated in Figure 7 and demonstrates that significantly better results were achieved with the MST orig. model. In short, the AARD values are up to three times lower when the MST orig. model is used instead of the PC-SAFT EoS.

### 2.6. Estimation of Dissolution, Sublimation, and Solvation Enthalpies

As noted in Section 2.1.2, the Chrastil and Bartle et al. models include an energy term that is the coefficient of temperature term. The dissolution enthalpy (${\Delta h}_{diss}$) is defined as the sum of solvation enthalpy (${\Delta h}_{solv}$) and sublimation enthalpy (${\Delta h}_{sub}$). According to Chrastil, the enthalpy of dissolution can be estimated from the parameter α in the Chrastil model by the following relationship: ${\Delta h}_{diss}=-\alpha \cdot R_{0}$, where $R_{0}$ is the universal gas constant. Furthermore, as suggested by Bartle et al., the enthalpy of sublimation can be estimated from the parameter $a_{2}$ in the Bartle ext. model by ${\Delta h}_{sub\;}=-a_{2}\cdot R_{0}$. Finally, the enthalpy of solvation can then be calculated from the difference between the dissolution enthalpy and the sublimation enthalpy. The estimated values of these three enthalpies for the precursors investigated are summarized in Table 7. From these data, the following trends are observed for ${\Delta h}_{diss}$ and ${\Delta h}_{sub}$: Cu(tmhd)_2_ > Pt(cod)me_2_ > Pd(acac)_2_ > Cu(acac)_2_ > Pd(tmhd)_2_ > Pt(acac)_2_. For the absolute values of ${\Delta h}_{solv}$ holds: Pd(cod)me_2_ > Pd(acac)_2_ > Cu(acac)_2_ = Cu(tmhd)_2_ > Pt(acac)_2_ > Pd(tmhd)_2_. However, the enthalpic properties derived should not be accepted unconditionally but should, at best, be seen as a rough estimate [25].

In general, from the data given in Table 7, it follows that precursor sublimation and solvation were endothermic and exothermic processes, respectively. Furthermore, the calculated solvation enthalpy indicates the presence of non-negligible intermolecular interactions between the precursors and CO_2_ molecules in the binary system.

## 3. Materials and Experimental Methods

### 3.1. Materials

In this paper, experimental determined solubility data for the metal precursors Cu(acac)_2_, Pd(acac)_2_, and Pt(acac)_2_ in scCO_2_ are reported. Detailed specifications of the substances investigated are listed in Table 8. The precursors were used as received. Usually, the experiments were performed at temperatures ranging from 313 to 353 K and pressures ranging from 10 to 40 MPa, respectively.

### 3.2. Experimental

The solubilities of the different precursors in scCO_2_ were determined using a static method [51] coupled with the gravimetric analysis described by Sherman et al. in detail [52]. As a rule, a considerable excess of precursor was weighed into an open stainless steel sample container using a precision scale (Mettler Toledo, Gießen, Germany; type XS 105 DU, ±0.02 mg) and loaded into the high-pressure view cell (SITEC-Sieber Engineering, Maur (Zurich), Switzerland; type 740.2027). Afterward, the view cell was evacuated, and with 2 silicone heating jackets (LCS Isotherm, Frankfurt a. M., Germany; 30 VAC, 60 Watt), heated to the target temperature using a temperature controller (Eurotherm, Limburg an der Lahn, Germany; type 2116). As soon as the target temperature was reached, the desired CO_2_ pressure was adjusted by a pressure generator (SITEC-Sieber Engineering, type 750.1100). For improved mixing of the supercritical mixture, a magnetic stir bar was placed inside the view cell. These conditions were held for 20 h to dissolve the precursor in scCO_2_. Thereafter, the view cell was slowly depressurized (Δp/Δt < 0.3 $MPa\cdot {min}^{-1}$), opened, and the sample container was reweighed. The difference between the initial and final mass of the precursor thus gave the amount of precursor dissolved in scCO_2_. The amount of CO_2_ filled into the view cell was determined by weighing the CO_2_ storage container with a scale (Mettler Toledo, PM 6100, ±0.02 g) before and after filling CO_2_ into the view cell. The uncertainty in solubility ${∆y}_{2}=y_{2,max}-y_{2,min}$ was calculated with the $y_{2,max}$ and $y_{2,min}$ determined with the uncertainties in mass determination (see above), using the respective precursor and CO_2_ masses.

The temperature in the high-pressure view cell was measured with a thermocouple (Thermocoax, Caligne, France; type K—NiCrNi) combined with a temperature indicator (Eurotherm, type 2116), which was calibrated beforehand against a Pt-25 thermometer (Anton Paar GmbH, Graz, Austria; type MKT-25, ±0.001 K) provided with a National Bureau of Standards certificate. The accuracy of the temperature measurement was within the total limit of ±0.2 K.

**Figure 8:**
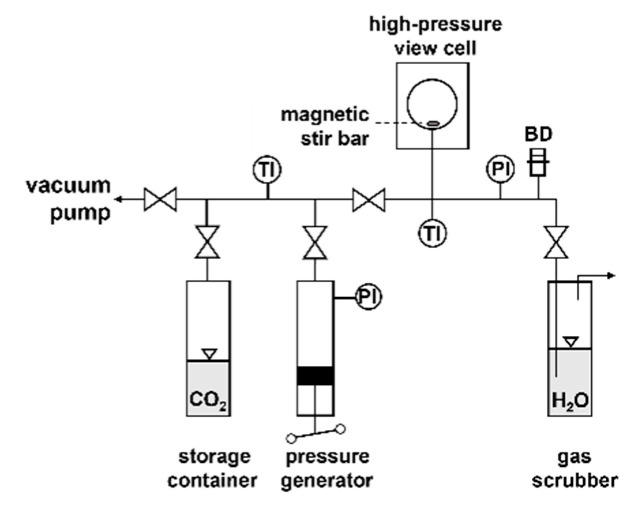
Scheme of the experimental setup (PI = pressure indicator, TI = temperature indicator, and BD = bursting disk).

A piezoresistive pressure transmitter (WIKA GmbH, Klingenberg, Germany; type S10) combined with a pressure indicator (WIKA GmbH, type Tronic Line), which were calibrated beforehand against a high-precision standard dead-weight gauge (Desgranges & Hout, DH Budenberg Huntington Beach, CA, USA; 5203 S, ±0.01 %), was used to measure the system pressure. The total uncertainty of the pressure measurement was within ±0.02 MPa. The uncertainties in the density (${\Delta \rho }_{1}/\rho_{1}$) resulting from the measurement uncertainties for temperature and pressure are listed in Table 1, Table 2 and Table 3. The uncertainty in density $∆\rho_{1}=\rho_{1,max}-\rho_{1,min}$ was calculated with $\rho_{1,max}$ and $\rho_{1,min}$ using the temperature and pressure uncertainties given above (Figure 8).

## 4. Conclusions

The solubilities of Cu(acac)_2_, Pd(acac)_2_, and Pt(acac)_2_ in scCO_2_ were measured at temperatures ranging from 313 to 353 K and pressures from 10 to 40 MPa. As a result, the solubility of the different (acac)_2_ precursors in scCO_2_ ranges from $y_{2}=$ 9·10^−6^ to 1.2·10^−4^ $mol\cdot {mol}^{-1}$ and increases as follows: Cu(acac)_2_ < Pt(acac)_2_ < Pd(acac)_2_. These data, together with the solubility data for Cu(tmhd)_2_, Pd(tmhd)_2_, and Pt(cod)me_2_ in CO_2_, partly reported earlier, and numerous data from the literature were correlated using semi-empirical density-based models, namely Chrastil, KJ ext., Bartle ext., and Méndez–Santiago–Teja mod. (MST) with three model parameters and the original MST model with two model parameters. Based on the AARD values, the order for the ability to correlate the solubility is: Chrastil > KJ ext. > MST mod. > Bartle ext. > MST orig.

Thus, Chrastil’s three-parameter model shows the best results, considering the fact that only density and temperature data are required for parameter adjustment. In addition, the quality of the data representation seems to decrease when, as in the case of Bartle ext. and MST mod., the pressure is included. From an engineering point of view, the simple density-based models provide sufficiently accurate results in the correlation of the experimental solubility data. Furthermore, the density-based models provided a significantly (by a factor of three) better fit of the solubility data than the PC-SAFT EoS. Finally, based on the Chrastil and Bartle ext. models, the dissolution, sublimation, and solvation enthalpies of the metal precursors investigated were derived.

## Figures and Tables

**Table 1:** Experimental solubility data of Cu(acac)_2_ in scCO_2_. The CO_2_ density was taken from the NIST Chemistry WebBook [24]; $y_{2}$ was calculated according to Equation (14).

T (K)	p (MPa)	$\rho_{1}$ $(mol\cdot {dm}^{-3}$)	${\Delta \rho }_{1}/\rho_{1}$ (%)	$y_{2}$ $(mol\cdot {mol}^{-1}$)	${\Delta y}_{2}$ $(mol\cdot {mol}^{-1}$)
333.1	15.23	13.92	0.87	1.05·10^−5^	±2.48·10^−6^
333.1	20.25	16.54	0.43	1.78·10^−5^	±2.26·10^−6^
333.1	25.16	17.91	0.30	2.05·10^−5^	±2.12·10^−6^
333.1	30.33	18.91	0.23	2.47·10^−5^	±2.03·10^−6^
333.1	35.52	19.68	0.19	2.99·10^−5^	±2.05·10^−6^
333.1	40.13	20.24	0.17	3.16·10^−5^	±2.02·10^−6^
353.2	15.14	9.84	1.01	9.66·10^−6^	±3.34·10^−6^
353.2	20.28	13.64	0.57	2.71·10^−5^	±2.79·10^−6^
353.2	25.21	15.66	0.37	3.62·10^−5^	±2.63·10^−6^
353.2	30.23	16.99	0.28	4.32·10^−5^	±2.54·10^−6^
353.2	35.46	18.00	0.22	4.98·10^−5^	±2.50·10^−6^
353.2	40.50	18.77	0.19	5.96·10^−5^	±2.55·10^−6^

**Table 2:** Experimental solubility data of Pd(acac)_2_ in scCO_2_. The CO_2_ density was taken from the NIST Chemistry WebBook [24]; $y_{2}$ was calculated according to Equation (14).

T (K)	p (MPa)	$\rho_{1}$ $(mol\cdot {dm}^{-3}$)	${\Delta \rho }_{1}/\rho_{1}$ (%)	$y_{2}$ $(mol\cdot {mol}^{-1}$)	${\Delta y}_{2}$ $(mol\cdot {mol}^{-1}$)
313.1	10.08	14.43	1.70	8.95·10^−6^	±1.71·10^−6^
313.1	12.62	16.68	0.66	1.54·10^−5^	±1.55·10^−6^
313.1	15.12	17.77	0.45	2.16·10^−5^	±1.54·10^−6^
313.1	20.10	19.10	0.30	2.58·10^−5^	±1.48·10^−6^
313.1	25.00	19.99	0.23	2.52·10^−5^	±1.38·10^−6^
313.1	30.01	20.68	0.19	2.30·10^−5^	±1.30·10^−6^
333.1	10.07	6.68	1.64	4.17·10^−6^	±3.18·10^−6^
333.1	12.58	10.87	1.66	9.65·10^−6^	±2.15·10^−6^
333.1	15.10	13.82	0.89	2.04·10^−5^	±1.92·10^−6^
333.1	20.04	16.47	0.43	4.16·10^−5^	±1.87·10^−6^
333.1	25.06	17.90	0.30	5.32·10^−5^	±1.86·10^−6^
333.1	29.89	18.84	0.24	5.57·10^−5^	±1.76·10^−6^
353.2	10.14	5.14	1.01	9.13·10^−6^	±4.21·10^−6^
353.2	12.53	7.24	1.08	1.46·10^−5^	±3.16·10^−6^
353.2	15.02	9.72	1.02	1.96·10^−5^	±2.52·10^−6^
353.2	20.06	13.52	0.59	4.58·10^−5^	±2.42·10^−6^
353.1	25.05	15.61	0.38	7.83·10^−5^	±2.46·10^−6^
353.3	29.83	16.89	0.28	9.92·10^−5^	±2.52·10^−6^

**Table 3:** Experimental solubility data of Pt(acac)_2_ in scCO_2_. The CO_2_ density was taken from the NIST Chemistry WebBook [24]; $y_{2}$ was calculated according to Equation (14).

T (K)	p (MPa)	$\rho_{1}$ $(mol\cdot {dm}^{-3}$)	${\Delta \rho }_{1}/\rho_{1}$ (%)	$y_{2}$ $(mol\cdot {mol}^{-1}$)	${\Delta y}_{2}$ $(mol\cdot {mol}^{-1}$)
313.2	15.15	17.78	0.45	1.37·10^−5^	±1.42·10^−6^
313.2	20.13	19.11	0.29	2.10·10^−5^	±1.43·10^−6^
313.2	25.15	20.00	0.23	2.36·10^−5^	±1.40·10^−6^
313.2	30.16	20.69	0.19	3.02·10^−5^	±1.44·10^−6^
313.2	35.02	21.24	0.17	2.98·10^−5^	±1.39·10^−6^
313.2	40.06	21.73	0.15	2.88·10^−5^	±1.34·10^−6^
333.1	15.10	13.82	0.89	1.23·10^−5^	±1.76·10^−6^
333.1	20.12	16.49	0.43	2.70·10^−5^	±1.73·10^−6^
333.1	25.26	17.94	0.29	3.95·10^−5^	±1.77·10^−6^
333.1	30.16	18.88	0.23	4.74·10^−5^	±1.79·10^−6^
333.1	35.08	19.62	0.20	5.73·10^−5^	±1.86·10^−6^
333.1	40.19	20.25	0.17	6.20·10^−5^	±1.85·10^−6^
353.2	15.20	9.90	1.00	7.34·10^−6^	±2.21·10^−6^
353.2	20.19	13.60	0.58	2.73·10^−5^	±2.04·10^−6^
353.2	25.20	15.65	0.37	4.30·10^−5^	±2.05·10^−6^
353.3	30.22	16.98	0.28	6.39·10^−5^	±2.21·10^−6^
353.2	35.26	17.97	0.22	8.80·10^−5^	±2.44·10^−6^
353.2	40.16	18.73	0.19	1.22·10^−4^	±2.82·10^−6^

**Table 4:** Overview over own and published solubility data for Cu(acac)_2_, Cu(tmhd)_2_, Pd(acac)_2_, Pd(tmhd)_2_, Pt(acac)_2_, and Pt(cod)me_2_ in scCO_2_.

	Isotherms	N	T (K)	p (MPa)	Ref.
Cu(acac)_2_	8	83	range: 308–353	range: 8–40	[overall]
	1	8	313	10–35	[44]
	3	15	308, 318, 328	10–30	[40]
	1	3	333	15–30	[45]
	1	13	313	8–30	[46]
	4	32	313, 323, 333, 343	14–30	[47]
	2	12	333, 353	15–40	[this work]
Cu(tmhd)_2_	6	62	range: 313–373	range: 10–35	[overall]
	1	8	313	10–35	[44]
	1	4	333	10–18	[45]
	4	35	313, 323, 333, 343	12–30	[47]
	1	7	333	10–17	[34]
	4	8	313, 333, 353, 373	11–15	[49]
Pd(acac)_2_	3	39	range: 313–353	range: 10–30	[overall]
	1	3	313	10–30	[50]
	1	3	333	15–30	[45]
	1	15	313	10–30	[46]
	3	18	313, 333, 353	10–30	[this work]
Pd(tmhd)_2_	3	18	323–343	15–40	[35]
Pt(acac)_2_	3	30	313–353	range: 10–40	[overall]
	1	5	313	10–30	[50]
	1	7	313	10–29	[46]
	3	18	313, 333, 353	15–40	[this work]
Pt(cod)me_2_	3	74	range: 313–353	range: 9–32	[overall]
	1	3	313	10–30	[50]
	3	54	313, 333, 353	9–30	[45]
	3	17	313, 333, 353	13–32	[34]

**Table 5:** Values were obtained by fitting the individual model parameters of Chrastil, KJ ext., Bartle ext., MST orig., and MST mod. to the experimental solubility data. $R_{adj}^{2}$ has been calculated according to Equation (12).

**Chrastil**	β	γ	α **(K)**	$R_{adj}^{2}$
Cu(acac)_2_	−6.71	3.03	−4176	0.892
Cu(tmhd)_2_	3.08	4.82	−7729	0.974
Pd(acac)_2_	−4.31	2.61	−4348	0.858
Pd(tmhd)_2_	−5.28	3.42	−3968	0.962
Pt(acac)_2_	−10.05	3.39	−3277	0.853
Pt(cod)me_2_ ^(a)^	−0.54	2.84	−4614	0.971
**KJ ext.**	$b_{0}^{⋆}$	$b_{1}^{⋆}$ $\left({dm}^{3}\cdot {mol}^{-1}\right)$	$b_{2}^{⋆}$ **(K)**	$R_{adj}^{2}$
Cu(acac)_2_	−0.88	0.22	−4486	0.910
Cu(tmhd)_2_	11.50	0.37	−8088	0.945
Pd(acac)_2_	0.49	0.23	−4779	0.928
Pd(tmhd)_2_	0.89	0.21	−4008	0.949
Pt(acac)_2_	−4.14	0.21	−3233	0.803
Pt(cod)me_2_ ^(a)^	4.63	0.21	−4832	0.982
**Bartle ext.**	$a_{0}$	$a_{1}$ $\left({dm}^{-3}\cdot {mol}^{-1}\right)$	$a_{2}$ **(K)**	$R_{adj}^{2}$
Cu(acac)_2_	13.75	0.35	−6461	0.951
Cu(tmhd)_2_	28.45	0.47	−10016	0.979
Pd(acac)_2_	15.85	0.34	−6911	0.956
Pd(tmhd)_2_	15.38	0.36	−5956	0.983
Pt(acac)_2_	10.89	0.36	−5390	0.952
Pt(cod)me_2_	20.88	0.35	−7400	0.969
**MST orig. ^(b)^**	A	B $\left({dm}^{3}\cdot {mol}^{-1}\right)$		$R_{adj}^{2}$
Cu(acac)_2_	1649	109.5	–	0.951
Cu(tmhd)_2_	2668	152.5	–	0.974
Pd(acac)_2_	2421	129.4	–	0.956
Pd(tmhd)_2_	2789	135.2	–	0.921
Pt(acac)_2_	1919	146.6	–	0.875
Pt(cod)me_2_	–	–	–	–
**MST mod.**	$A^{⋆}$ **(K)**	$B^{⋆}$ $\left(K\cdot {dm}^{3}\cdot {mol}^{-1}\right)$	$C^{⋆}$	$R_{adj}^{2}$
Cu(acac)_2_	−8325	111.5	11.72	0.954
Cu(tmhd)_2_	−12413	155.8	25.86	0.976
Pd(acac)_2_	−8755	114.8	13.55	0.968
Pd(tmhd)_2_	−8135	119.7	13.89	0.987
Pt(acac)_2_	−7400	118.1	8.97	0.943
Pt(cod)me_2_	−9273	117.4	18.56	0.975

^(a)^ Parameters determined after the consistency test (see Figure 3). ^(b)^ Associated Clausius–Clapeyron parameters of Equation (6) are listed in Table A1 in Appendix A.

**Table 6:** Summary of modeling the solubility $y_{2}$ in scCO_2_ using different models. The AARD values have been calculated according to Equation (13).

	N	AARD (%)					Ref.
		Chrastil	KJ Ext.	Bartle Ext.	MST Orig.	MST Mod.	
Cu(acac)_2_	83	14.3	13.1	15.7	15.0	14.8	[overall]
	8	13.4	12.2	14.9	11.4	13.9	[44]
	15	18.0	13.0	21.0	23.4	20.5	[40]
	3	12.5	9.3	9.2	6.4	8.4	[45]
	13	22.4	18.9	21.8	21.0	20.8	[46]
	32	8.5	8.2	9.8	11.3	9.6	[47]
	12	17.8	21.9	20.4	12.3	16.9	[this work]
Cu(tmhd)_2_	62	15.6	26.6	19.3	21.2	20.5	[overall]
	8	44.1	57.1	45.8	60.6	51.6	[44]
	4	20.4	60.8	44.7	47.2	44.6	[45]
	35	9.7	16.5	11.9	10.8	11.0	[47]
	7	11.3	24.6	13.9	16.7	15.1	[34]
	8	14.2	24.5	16.7	18.0	24.0	[49]
Pd(acac)_2_	39	26.6	20.3	23.7	28.6	21.1	[overall]
	3	33.0	29.5	25.6	12.5	24.7	[50]
	3	16.1	10.0	14.7	30.8	9.8	[45]
	15	33.1	24.5	28.7	26.3	25.7	[46]
	18	21.8	16.9	20.7	32.9	18.6	[this work]
Pd(tmhd)_2_	18	8.0	9.0	8.5	24.4	7.7	[35]
Pt(acac)_2_	30	18.7	21.2	16.5	35.4	18.6	[overall]
	5	12.8	13.6	12.3	33.2	12.4	[50]
	7	17.2	18.3	14.3	36.6	14.5	[46]
	18	20.9	24.5	18.6	35.5	21.8	[this work]
Pt(cod)me_2_	74	8.7 ^(c)^	8.9 ^(c)^	17.9	–	15.8	[overall]
	3	10.5 ^(c)^	20.1 ^(c)^	71.9	–	65.3	[50]
	54	6.6 ^(c)^	7.7 ^(c)^	15.7	–	13.7	[45]
	17	14.4 ^(c)^	11.4 ^(c)^	15.4	–	13.7	[34]

^(c)^ AARD values determined after the test of consistency (see Figure 3).

**Table 7:** Estimated dissolution (${\Delta h}_{diss}$), sublimation (${\Delta h}_{sub}$), and solvation (${\Delta h}_{solv}$) enthalpies for the precursors investigated.

	${\Delta h}_{diss}$ (Chrastil) $(kJ\cdot {mol}^{-1}$)	${\Delta h}_{sub}$ (Bartle Ext.) $(kJ\cdot {mol}^{-1}$)	${\Delta h}_{solv}$ $(kJ\cdot {mol}^{-1}$)
Cu(acac)_2_	34.7	53.7	−19.0
Cu(tmhd)_2_	64.3	83.3	−19.0
Pd(acac)_2_	36.1	57.5	−21.4
Pd(tmhd)_2_	33.0	49.5	−16.5
Pt(acac)_2_	27.2	44.8	−17.6
Pt(cod)me_2_	38.4	61.5	−23.1

**Table 8:** Specifications of the substances used for the solubility measurements.

	CAS	M $(g\cdot {mol}^{-1}$)	Purity (%)	Supplier
Cu(acac)_2_	13395-16-9	261.78	98	abcr GmbH ^(d)^
Pd(acac)_2_	14024-61-4	304.64	99	abcr GmbH ^(d)^
Pt(acac)_2_	15170-57-7	393.29	98	abcr GmbH ^(d)^
CO_2_	124-38-9	44.01	99.995	Air Liquide ^(e)^

^(d)^ Karlsruhe, Germany; ^(e)^ Düsseldorf, Germany.

## Data Availability

The data that support the findings of this study are available from the corresponding author upon reasonable request.

## Abbreviations

The following abbreviations are used in this manuscript:

Abbreviation	unit	
AARD	%	average absolute relative deviation
acac		acetylacetonate
calc		calculated
CAS		Chemical Abstracts Service
cod		cyclooctadiene
${\Delta h}_{solv}$	$kJ\cdot {mol}^{-1}$	solvation enthalpy
${\Delta h}_{sub}$	$kJ\cdot {mol}^{-1}$	sublimation enthalpy
${\Delta h}_{diss}$	$kJ\cdot {mol}^{-1}$	dissolution enthalpy
E		enhancement factor
EoS		equation of state
exp		experimental
ext.		extended
GCM		group contribution method
K		number of adjustable parameters in the correlation model
KJ		Kumar–Johnston
M	$g\cdot {mol}^{-1}$	molar mass
m	g	mass
me		methyl
mod.		modified
MST		Méndez–Santiago–Teja
N		number of data points
$n_{1}$	mole	mole CO_2_
$n_{2}$	mole	mole precursor
NP		nanoparticle
orig.		original
p	MPa	pressure
$p_{0}$	Pa	=1 Pa, Equation (6)
$p_{\#}$	MPa	=1 MPa, Equation (7)
$p_{ref}$	MPa	=0.1 MPa, Equation (3)
$p_{2,sub}$	Pa	sublimation pressure of the solid
PC-SAFT		perturbed-chain statistical associating fluid theory
PR		Peng–Robinson
$R_{0}$	$J\cdot {mol}^{-1}\cdot K^{-1}$	$\mathrm{universal}\;\mathrm{gas}\;\mathrm{constant}\;(=8.314\;J\cdot {mol}^{-1}\cdot K^{-1}$)
$R^{2}$		squared correlation coefficient
$R_{adj}^{2}$		adjusted squared correlation coefficient
$\rho_{0}$	$mol\cdot {dm}^{-3}$	$=1\;mol\cdot {dm}^{-3}$
$\rho_{1}$	$mol\cdot {dm}^{-3}$	density of CO_2_
$\rho_{c}$	$mol\cdot {dm}^{-3}$	$=10.5\;mol\cdot {dm}^{-3}$
$\rho_{ref}$	$mol\cdot {dm}^{-3}$	$=15.90\;mol\cdot {dm}^{-3}$, Equation (3)
sc		supercritical
SCF		supercritical fluid
SFRD		supercritical fluid reactive deposition
T	K	temperature
t	min	time
tmhd		tetramethyl-3,5-heptandione
$y_{2}$	$mol\cdot {mol}^{-1}$	precursor solubility
Parameters…		
α, β, γ		parameters of the Chrastil model, Equation (1)
$b_{0\;}^{⋆},b_{1\;}^{⋆},b_{2\;}^{⋆}$		parameters of the KJ ext. model, Equation (2)
$a_{0},\;a_{1},\;a_{2}$		parameters of the Bartle ext. model, Equation (3)
A,B		parameters of the MST orig. model, Equation (4)
a, b		parameters of the Clausius-Clapeyron equation, Equation (6)
$A^{⋆},B^{⋆},\;C^{⋆}$		parameters of the MST mod. model, Equation (7)

## Appendix A. Sublimation Pressures

Parameters of the Clausius–Clapeyron equation for the different sublimation pressures used in the present investigation, together with the temperature range over which the data were determined, are listed in Table A1 [37,41,48,53,54,55,56].

**Table A1:** Parameters for Equation (6) and temperature range over which the data were determined.

		T Range	a	b (K)	Ref.
Cu(acac)_2_	Nasebulin	353–477 K	24.5	−9603	[37]
Cu(tmhd)_2_	Tobaly, Ribeiro, Siddiqi	362–452 K	37.7	−14,392	[41,53,54]
Pd(acac)_2_	Morozova	347–453 K	33.6	−13,425	[55]
Pd(tmhd)_2_	Igumenov	348–398 K	27.9	−15,106	[56]
Pt(acac)_2_	Morozova	332–453 K	31.7	−12,737	[55]
Pt(cod)me_2_	Hierso	333–353 K	17.2	−5441	[48]
